# Effect of the Dynamic Neuromuscular Stabilization Technique on Functional Capacity in Overweight and Obese Individuals: A Randomized Controlled Trial

**DOI:** 10.7759/cureus.42076

**Published:** 2023-07-18

**Authors:** Purva Gulrandhe, Rakesh K Kovela, Snehal Samal

**Affiliations:** 1 Physiotherapy, Ravi Nair Physiotherapy College, Datta Meghe Institute of Medical Sciences (Deemed to be University), Wardha, IND; 2 Physiotherapy, Nitte Institute of Physiotherapy (Deemed to be University), Mangalore, IND; 3 Neurophysiotherapy, Ravi Nair Physiotherapy College, Datta Meghe Institute of Medical Sciences (Deemed to be University), Wardha, IND

**Keywords:** bmi, rehabilitation, overweight, obesity, manual therapy, dynamic neuromuscular stabilization

## Abstract

Background

Individuals with overweight and obesity (OW/OB) have poor performance in weight-bearing exercises, resulting in low functional capacity. The dynamic neuromuscular stabilization (DNS) technique was created to engage the core-postural chain in conjunction with the core muscles, generating enough intra-abdominal pressure to dynamically support the spine through muscular activity. DNS is a strategy that emphasizes the activation of the spine's intrinsic stabilizers, as well as proper breathing patterns, before any intended functional movement. The aim and objective of this study was to determine the effectiveness of the DNS technique on functional capacity in OW/OB individuals and to compare the effectiveness of the DNS and conventional approach.

Methods

The study recruited 100 individuals, who were separated into an experimental group (DNS technique) and a control group (conventional exercises), each with 50 participants. Outcome measures, including a six-minute walk test (SMW test) and body mass index (BMI), were taken pre-treatment and post-treatment.

Results and discussion

Based on the inclusion criteria of this study, the age group included was 20 to 25 years old. Pre- and post-treatment statistically significant changes were observed in the experimental and control groups in the BMI and SMW test. However, the BMI (kg/m^2^) was not statistically significant in the experimental group (t-value=-0.15, p=0.87) and control group (t-value=-0.22, p=0.82). Moreover, in the SMW test (meter), no statistical significance was found in the experimental group (t-value=-0.15, p=0.87) and control group (t-value=- 0.22, p=0.82).

Conclusions

Both groups are effective in increasing the functional capacity of obese and overweight individuals. The study indicates a strong need for further research into its long-term effectiveness in the OW/OB population.

## Introduction

Obesity (OB) is a major concern for community health across the globe. A body mass index (BMI) between 25 kg/m² and 29.9 kg/m² is considered overweight, whereas a BMI of 30 kg/m² or higher is considered obese. [1-3]. OB affects more than 13.5 crore people in India. OB prevalence in India varies from rural to urban and state to state [4,5]. OB is a chronic condition that comes with several complications that impact a variety of body processes [1]. OB is linked to functional limitations in physical performance and an increased risk of developing functional disabilities [6]. The capacity for physical activity is a measure of physical fitness and includes numerous elements (e.g., cardiopulmonary, speed-agility, and muscular fitness), all of which are linked to a person's overall health. OW/OB is adversely connected to all of these elements, which may have poor health outcomes [4]. Obese people appear weaker after sudden weight loss. This relative weakness may be caused by neurological alterations, decreased mobility, and changes in muscle architecture [6,7]. Even in the absence of weight loss, regular workouts can ameliorate the health implications of OB. Overall, exercise is an essential, although frequently neglected, tool in the control of OB [8]. OB management is centered on lifestyle modification [9]. A person with OW/OB is 1.5 times more likely than their healthy-weight counterparts to display an abnormal body posture, which is defined as body segments in non-optimal positioning, resulting in mechanical stress and overactivation of muscles [10]. Individuals with OW/OB have poorer performance in weight-bearing exercises, such as running and walking, and reduced cardiorespiratory fitness, resulting in lower functional capacity [11]. In individuals with OB, the adipose tissue surrounding the abdomen, rib cage, and visceral cavity can impose additional strain on the chest wall and lead to a decrease in the functional residual capacity (FRC) [12].

Dynamic neuromuscular stabilization (DNS) approaches evaluate and stimulate local spinal stabilizers to enhance postural and respiratory efficiency for both prevention and intervention [13]. The DNS technique was created to engage the core-postural chain in conjunction with the core muscles, generating enough intra-abdominal pressure (IAP) to dynamically support the spine through muscular activities [14]. The optimal coordination of the diaphragm, pelvic floor, and abdominal muscles to govern IAP is required for optimal spinal stabilization. IAP works with the lumbar paraspinal muscles to provide dorsal spinal stability, minimizes compressive stresses on the spine, and offers ventral spinal stabilization [15].

DNS is a developing idea in the rehabilitation field that was developed by Professor Pavel Kolar after being influenced by Vojta's work on reflex locomotion. The idea of reflex locomotion describes how firm pressure stimulation across certain muscle areas causes distinct involuntary motor response patterns. These fundamental movement patterns have been called "global patterns." Activating peripheral regions or zones can trigger the neural circuitry that drives these intricate developmental processes [16]. The DNS rehabilitative technique helps in improving movement control that is focused on the scientific concepts of developmental kinesiology [17]. According to Kobesova and Kolar, there are three levels of sensorimotor control: a) brainstem and spinal level, b) cortical level, and c) subcortical level [16]. According to DNS, from a neurodevelopmental viewpoint, posture, pattern of breathing, and centration of joints (a posture that affects the joints to be in maximal congruency) should be investigated and managed [17]. To date, there is a dearth in the literature that emphasizes the use of the DNS concept in OW/OB individuals, where the effective core stands as a targeted structural component for functional capacity. As a result, there is a need to study the effect of the DNS technique on the functional capacity of individuals with OW/OB. This study aims to determine the effectiveness of the DNS technique in improving functional capacity among OW/OB individuals. The specific objectives were to evaluate the impact of the DNS technique on the functional capacity of OW individuals, to assess the effectiveness of the DNS technique in enhancing the functional capacity of OB individuals, and to compare the effectiveness of the DNS technique with conventional exercises in improving the functional capacity of both OW and OB individuals.

## Materials and methods

The study was conducted in various colleges of Datta Meghe Institute of Medical Sciences,Maharashtra, India, and the outpatient department (OPD) of Ravi Nair Physiotherapy College, Maharashtra, India, using a randomized controlled trial (RCT) design. It is an experimental study with an intervention approach. The targeted population included individuals of either gender, aged between 18 and 25 years, who were classified as OW and OB. The study spanned over a period of six months and employed probability sampling through the simple random sampling technique.

The sample size for the study was calculated using the following formula, resulting in 50 participants in each group, making a total of 100 participants.

$n=\frac{Za^{2}/2 .p.(1-p)}{d^{2}}$,

Where

Zα=the level of significance at 5%, i.e., 95% confidence interval=1.96

p=prevalence of obesity=6.8%=0.068

d=desired error of margin=7%=0.07

$n=\frac{1.96^{2}\times 0.068\times (1-0.068)}{0.07^{2}}$

n=49.68

n=50 patients in each group

The inclusion criteria for the participants were set to include individuals aged between 20 and 25 years who were willing to participate as volunteers and had a BMI ranging from 25 to 30 kg/m2. The exclusion criteria comprised individuals who had a history of fracture in the past six months, recent surgery, or fixed spinal deformity. For the study, certain materials were used, including a measuring tape, pulse oximeter, stopwatch, sphygmomanometer, and weighing machine.

**Figure 1:**
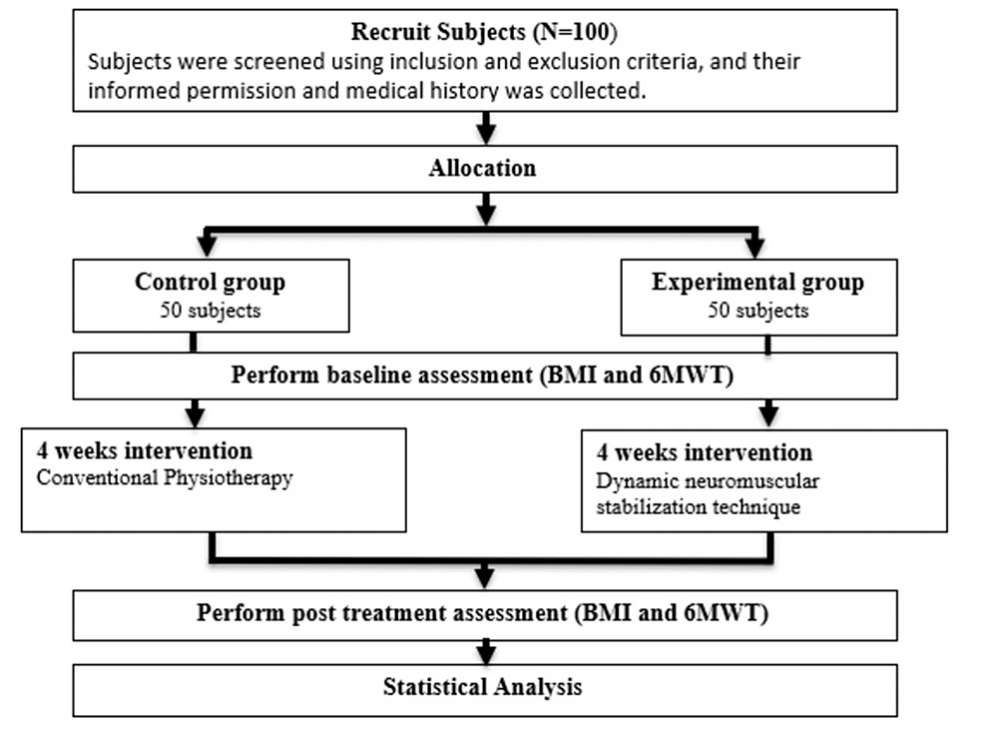
Allocation of samples in the two groups

The procedure involved sharing information about the study with various colleges of the university once ethical approval was granted. Interested individuals meeting the inclusion criteria were recruited and divided into two groups: the experimental (DNS) group and the control (conventional physiotherapy) group, using the sequentially numbered, opaque sealed envelopes (SNOSE) technique, explained in Figure 1. To ensure data integrity, the trial's data were securely stored in a closed facility with limited access, and subsequent evaluation was conducted by a biostatistician, in collaboration with the lead researcher. The therapy sessions were planned for 45 minutes a day, five days a week, for a duration of four weeks. Outcome measures were taken both before and after the treatment intervention. The study utilized the six-minute walk (SMW) test to measure functional capacity, along with the BMI as a standardized measure to identify anthropometric height/weight characteristics.

Control group

The conventional exercise prescription for the OW/OB therapy program was developed in accordance with the American College of Sports Medicine (ACSM) guidelines [18] and the frequency, intensity, time, type (FITT) principle. To enhance respiratory capacity, the therapy was started with 10 repetitions of breathing techniques. Stretching the gastrocnemius, soleus, thoracolumbar fascia (TLF), hip flexor, bicep femoris, and latissimus dorsii for 30 seconds with two repetitions was included in the warm-up exercises. Exercises included inclined prone isometric abdominals, curl-ups, walking, hip roll, bridging, hand-to-knee, and standing rotation, with two sets of eight repetitions each. Aerobic training included cycling and walking for 10 minutes, each followed by cool-down exercises. Cool-down exercises included stretching for the shoulder, triceps, quadriceps, toe touch, and overhead stretch for a 30-second hold for two repetitions.

Experimental group

**Figure 2:**
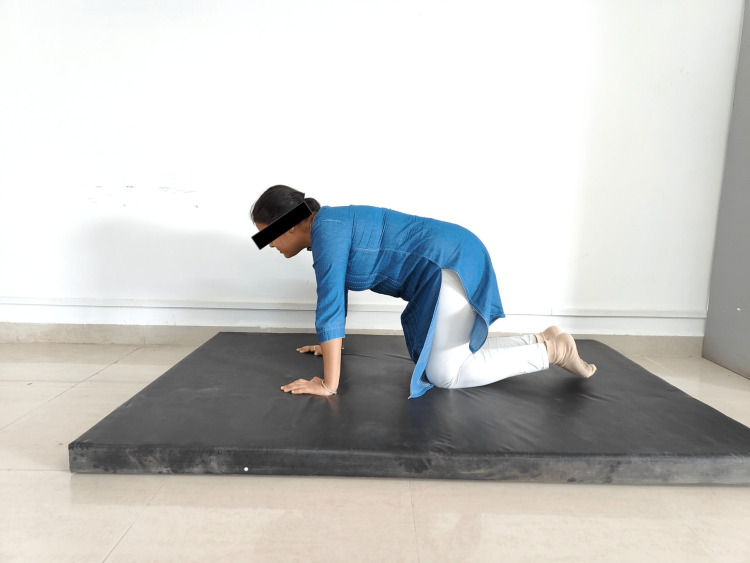
Participant performing DNS eight-month quadruped position DNS: dynamic neuromuscular stabilization

**Figure 3:**
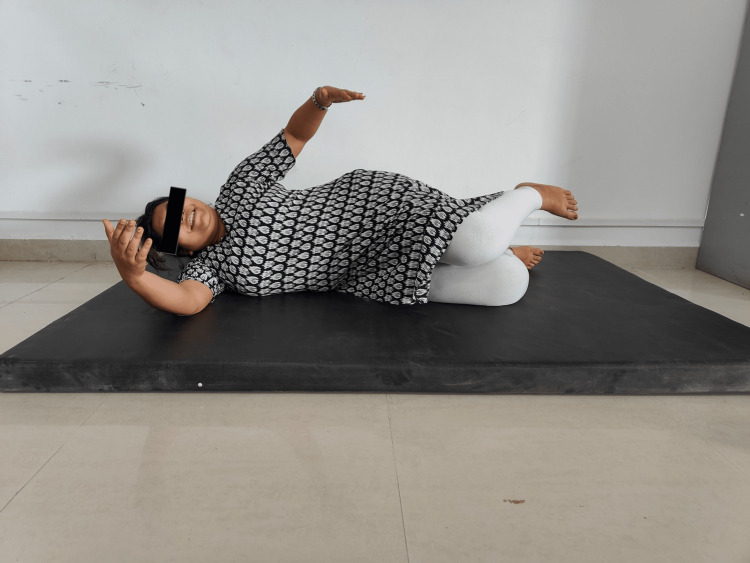
Participant performing DNS side-lying five-month position DNS: dynamic neuromuscular stabilization

The exercise prescription for the OW/OB treatment protocol was planned according to the ACSM standards based on the FITT principle. Initially, rehabilitation aimed to concentrate on the trunk and integrated spinal stability. The first focus was on retraining the respiratory function and integrating breathing and stabilization, followed by positions. Initially, a neutral chest position and correct breathing posture, as well as patient education, were planned. Every exercise and the following stage and exercise were based on a basis of each posture or developmental stage. The supine three-month position (a 90-degree hip flexion, functional centration at the hips, spine upright, shoulder girdles relaxed, and chest and pelvis properly aligned) and the prone three-month position (the upper arms are at a 90⁰ angle to the torso; support is on the elbows, anterior superior iliac spines and pubic symphysis, the wrist is in neutral avoiding ulnar deviation, patient learned to elongate/upright the spine, to keep the pelvis in neutral and avoid hyperextension in the cervical spine) were repeated a minimum of three times and stopped if there is any variation from the ideal pattern. Thereafter, two sets of eight repetitions were administered on each side laying in a five-month position (torso perpendicular to the table, the lower shoulder (support) and elbow are flexed to 90⁰ each, and the wrist in neutral and down side leg (support) is semi-flexed at the knee, with the hip and heel in line with the ischial tuberosity with the spine elongated and the pelvis in neutral) and seven-month oblique sitting position (on their side with support on the down-side forearm and hand, hip, thigh, and ankle, supporting shoulder was centrated with an active pressure onto the supporting forearm and hand while the supporting leg should actively press down and “out” to stabilize the pelvis in neutral). Exercise progression proceeded with resistance from exercise bands. Lastly, for eight months, all four (quadruped) positions (knees directly under the hips and hands and wrists directly under the shoulders with the spine elongated and the pelvis in neutral) were given, and repetitions were discontinued as soon as any deviation occurred. To progress this exercise, the individual was instructed to lift one arm (and the opposing leg) and hold it in place while maintaining proper joint centration and core stabilization. Figures 2 and 3 show patients performing the DNS technique.

Ethical approval was obtained from the Ethical Committee with reference number DMIMS(DU)/IEC/2022/1038.

Statistical analysis

Descriptive and inferential statistics, including the chi-square test, student's paired test, and unpaired t-test, and the software versions of IBM SPSS Statistics for Windows, Version 27 (Released 2020; IBM Corp., Armonk, New York, United States) and GraphPad Prism version 7.0 for Windows (GraphPad Software, Boston, Massachusetts, USA) were used for the statistical analysis, with p<0.05 considered as statistically significant.

## Results

**Figure 4:**
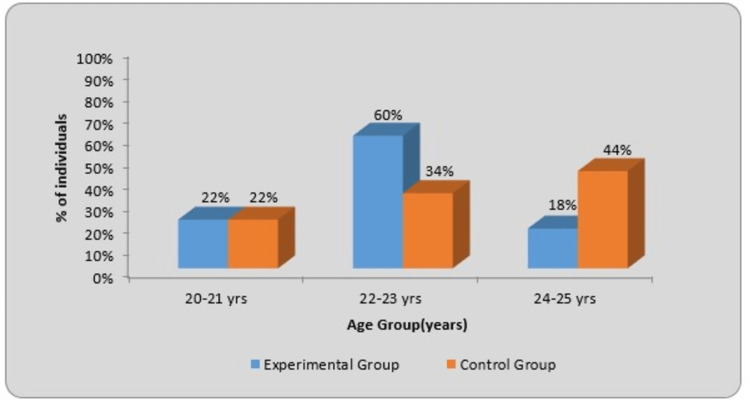
Graphical representation of age-wise (years) distribution of individuals in the two groups

According to the inclusion criteria, the age group included was 20 to 25 years. Table 1 shows the age-wise (years) distribution. The mean age in the experimental group was 22.38±1.30, with 11 participants (22%) between the ages of 20 and 21, 30 individuals (60%) between the ages of 22 and 23, and nine individuals (18%) between the ages of 24 and 25. The mean age in the control group was 23.08±1.72, with 11 individuals (22%) between the ages of 20 and 21, 17 individuals (34%) between the ages of 22 and 23, and 22 individuals (44%) between the ages of 24 and 25. In the experimental and control groups, the χ2 value (chi-square test) was 9.04, and p=0.010, which is statistically significant. The graphical representation of age (years) is given in Figure 4, in which the blue color indicates the experimental group and the orange color shows the control group.

**Table 1:** Age-wise (years) distribution of individuals in the two groups SD: standard deviation; S: statistically significant

Age in years	Experimental group	Control group	χ2 value
20-21 years	11 (22%)	11 (22%)	9.04; p=0.010, S
22-23 years	30 (60%)	17 (34%)	
24-25 years	9 (18%)	22 (44%)	
Total	50 (100%)	50 (100%)	
Mean±SD	22.38±1.30	23.08±1.72	
Range	20-25 years	20-25 years	

**Figure 5:**
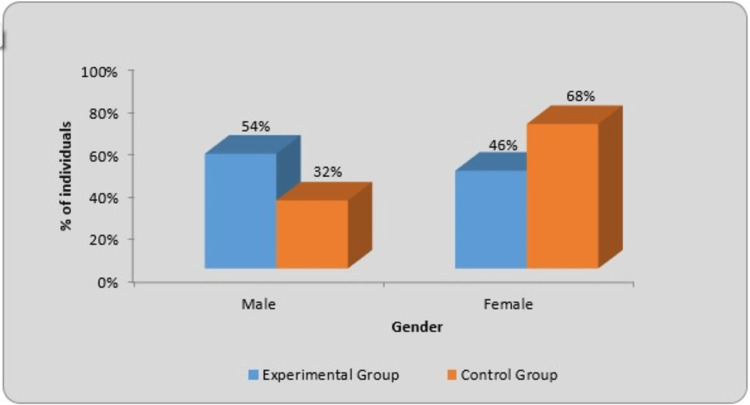
Graphical representation of gender-wise distribution of individuals in the two groups

In the experimental group, 27 male participants (54%) and 23 female (46%) participants were included. In the control group, 16 male participants (32%) and 34 female participants (68%) were included. In the experimental and control groups, the χ2​​​​​​​ value was 4.93 and p=0.026, which is statistically significant. The graphical representation of gender is given in Figure 5, in which the blue color indicates the experimental group and the orange color shows the control group. Table 2 shows the gender-wise distribution of participants.

**Table 2:** Gender-wise distribution of individuals in the two groups S: statistically significant

Gender	Experimental group	Control group	χ2 value
Male	27 (54%)	16 (32%)	4.93; p=0.026, S
Female	23 (46%)	34 (68%)	
Total	50 (100%)	50 (100%)	

**Figure 6:**
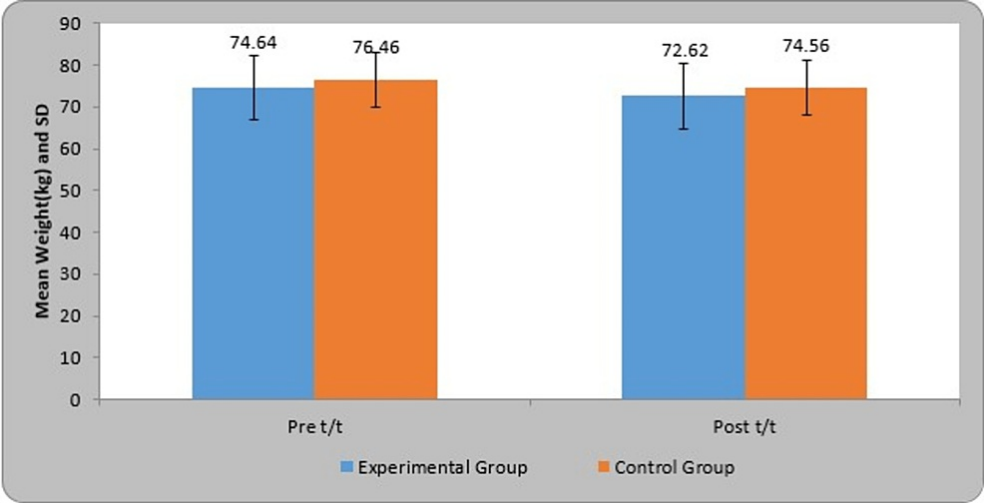
Graphical representation of the pre- and post-treatment comparison of the weights (kg) in the two groups SD: standard deviation

The pre- and post-treatment comparison of weight (kg) is given in Table 3. In the experimental group, the mean weight (kg) pre- and post-treatment was 74.64±7.77 and 72.62±7.94, respectively. The mean difference in weight (kg) in the experimental group was 2.02±0.95. In the control group, the mean weight (kg) pre- and post-treatment was 76.46±6.58 and 74.56±6.48, respectively. The mean difference in weight (kg) in the control group was 1.90±0.76. A statistically significant difference in weight (kg) between the experimental group and control group was discovered using Student's paired t-test (t-value=14.90, p=0.0001; t-value=17.61, p=0.0001). In comparison, the t-value using Student's unpaired t-test was found to be not statistically significant in the control group (t-value=1.33, p=0.18) and experimental group (t-value=1.26, p=0.21). Figure 6 shows the graphical representation of the pre- and post-treatment comparison of the weights.

**Table 3:** Pre- and post-treatment comparison of the weights (kg) in the two groups S: statistically significant; NS: not statistically significant

Group	Pre-treatment	Post-treatment	Mean difference	Student's paired t-test t-value
Experimental group	74.64±7.77	72.62±7.94	2.02±0.95	14.90; p=0.0001, S
Control group	76.46±6.58	74.56±6.48	1.90±0.76	17.61; p=0.0001, S
Comparison between the two groups (Student's unpaired t-test)				
t-value	1.26; p=0.21, NS	1.33; p=0.18, NS		

**Figure 7:**
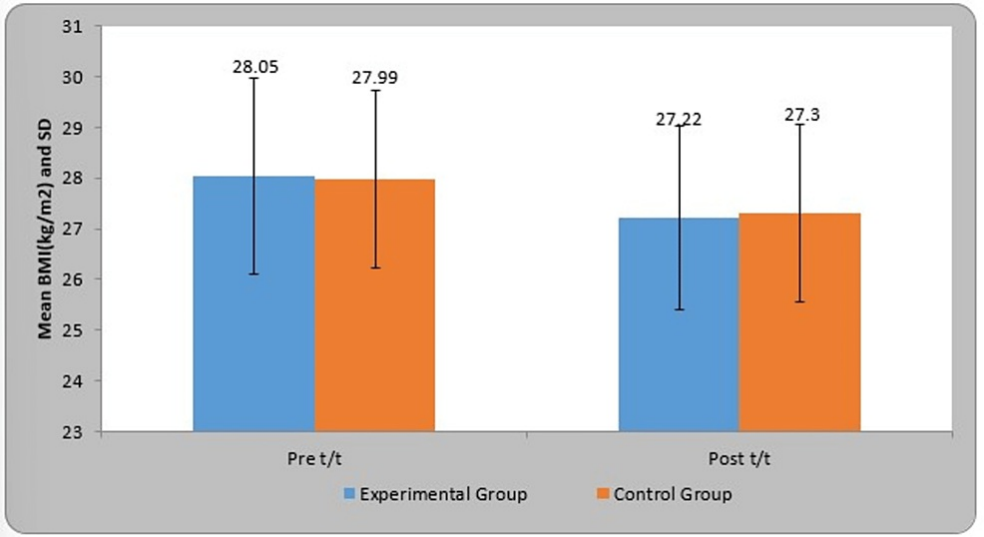
Graphical representation of the pre- and post-treatment comparison of BMI (kg/m2) in the two groups BMI: body mass index; SD: standard deviation

Table 4 shows the pre- and post-treatment comparison of BMI (kg/m2). In the experimental group, the mean BMI (kg/m2) pre- and post-treatment was 28.05±1.93 and 27.22±1.81, respectively. The mean difference in BMI (kg/m2) in the experimental group was 0.82±0.72. In the control group, the mean BMI (kg/m2) pre- and post-treatment was 27.99±1.75 and 27.30±1.75, respectively. The mean difference in the BMI (kg/m2) in the control group was 0.69±0.28. By using Student's paired t-test, statistically significant difference was found in the BMI (kg/m2) in the experimental group (t-value=8.10, p=0.0001) and control group (t-value=16.94, p=0.0001). In comparison, the t-value using Student's unpaired t-test was found to be not statistically significant in the control group (t-value=0.22, p=0.82) and experimental group (t-value=0.15, p=0.87). A graphical representation of the pre- and post-treatment comparison between the two groups is given in Figure 7.

**Table 4:** Pre- and post-treatment comparison of BMI (kg/m2) in the two groups BMI: body mass index

Group	Pre-treatment	Post-treatment	Mean difference	Student's paired t-test t-value
Experimental group	28.05±1.93	27.22±1.81	0.82±0.72	8.10; p=0.0001, S
Control group	27.99±1.75	27.30±1.75	0.69±0.28	16.94; p=0.0001, S
Comparison between the two groups (Student's unpaired t-test)				
t-value	0.15; p=0.87, NS	0.22; p=0.82, NS		

**Figure 8:**
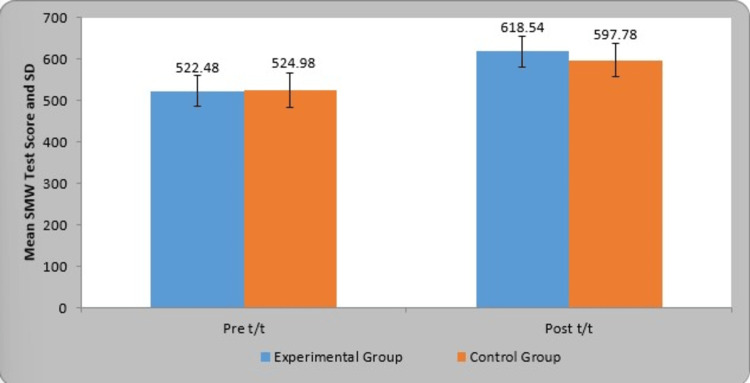
Graphical representation of the pre- and post-treatment comparison of the SMW test results (meter) in the two groups SMW: six-minute walk; SD: standard deviation

Table 5 represents the pre- and post-treatment comparison of the SMW test (meter) in the two groups. In the experimental group, the mean SMW test (meter) pre- and post-treatment was 522.48±36.98 and 618.54±36.90, respectively. The mean difference of the SMW test (meter) in the experimental group was 96.06±22.46. In the control group, the mean SMW test (meter) pre- and post-treatment was 524.98±41.83 and 597.78±40.12, respectively. The mean difference of the SMW test (meter) in the control group was 72.80±12.50. By using Student's paired t-test, a statistically significant difference was found in the SMW test (meter) in the experimental group (t-value=30.24, p=0.0001) and control group (t-value=41.15, p=0.0001). In comparison, the t-value using Student's unpaired t-test was found to be not statistically significant in the control group (t-value=0.22, p=0.82) and experimental group (t-value=0.15, p=0.87). The graphical representation of the pre- and post-treatment comparison of the SMW test (meter) in the two groups is given in Figure 8.

**Table 5:** Pre- and post-treatment comparison of the SMW test (meter) in the two groups SMW: six-minute walk; S: statistically significant; NS: not statistically significant

Group	Pre-treatment	Post-treatment	Mean difference	Student's paired t-test t-value
Experimental group	522.48±36.98	618.54±36.90	96.06±22.46	30.24; p=0.0001, S
Control group	524.98±41.83	597.78±40.12	72.80±12.50	41.15; p=0.0001, S
Comparison between two groups (Student's unpaired t-test)				
t-value	0.15; p=0.87, NS	0.22; p=0.82, NS		

## Discussion

The concepts of reflex-mediated core stability and neurodevelopmental kinesiology served as the foundation for DNS development. Musculoskeletal and neurological disorders are treated with DNS. OB is a major concern nowadays, which affects all vital systems, including cardiovascular, respiratory, and musculoskeletal. OB raises the likelihood of encountering a functional disability, encompassing challenges in mobility, posture, strength, balance, and muscle performance limitations. Our study aimed to find out the effect of DNS on OW/OB individuals' functional capacity. The gender-wise distribution in the experimental and control group was shown to be statistically significant (χ2=4.93, p=0.026). The age group selected for our study was 20 to 25 years. The age-wise distribution in the experimental and control group was statistically significant (χ2​​​​​​​=9.04, P=0.010). In comparison, using Student's unpaired t-test, a difference in the weight was found, but it was not statistically significant in the experimental group (t-value=1.26, p=0.21) and control group (t-value=1.33, p=0.18). Descriptive analysis was performed to keep both groups unbiased.

Yoon et al. conducted a study on DNS in stroke patients’ ultrasound imaging and electromyography processing. Their findings offered that patients with hemiparetic stroke showed more beneficial therapeutic advantages of DNS than normal control group [19]. Bae et al. conducted a study on DNS in patients with forward head posture (FHP) to evaluate its effects on the respiratory function with standard neck stabilization exercise, extensor strengthening exercises, and neck stretching. They discovered that after six weeks, the DNS group had the highest mean force vital capacity value. The DNS group had the highest changes in forced expiratory volume in one second [20].

Kim et al. examined the effectiveness of DNS training on balance in adolescent with spastic hemiparetic cerebral palsy for four weeks of training. The findings revealed that scores on the 10-meter walk test, the balance subtest of the Bruininks-Oseretsky Test of Motor Proficiency, and the SMW test all improved following the treatment compared to the initial value, and hence, it is considered a successful therapeutic strategy [21]. In our study, the SMW test was improved post-intervention when treated with DNS in OW/OB individuals.

According to a study by Cavaggioni et al., the benefits of core stabilization training based on abdominal fitness, diaphragmatic breathing, and movement efficiency were examined. The findings showed that while breathing exercises and stretching of the muscle chain can both improve pulmonary function, these effects are enhanced. Core stabilization is more efficient at enhancing abdominal fitness and pulmonary function than conventional abdominal exercises [22]. Breathing exercises should be considered an important aspect to gain more benefits.

In a research by Mousavi et al., the impact of central stability and the DNS approach on pain, abdominal muscle strength, hamstring muscle flexibility, balance, and quality of life (QoL) in males with persistent low back pain (LBP) were compared. Both training protocols considerably enhanced the variables of pain, static balance, and abdominal muscle strength. The QoL was also greatly enhanced by the DNS exercise, while the hamstring flexibility was significantly increased by the central stability exercise [23]. Ghavipanje et al. conducted a research on the effectiveness of DNS training in postpartum obese women having LBP for six weeks, and 40 postpartum obese women with LBP were randomly allocated to either DNS or conventional treatment six times a week. They concluded that DNS is therapeutically recommended according to optimum patterns, which are ontogenetic to provide optimal outcomes for postpartum obese women having LBP [24]. 

Nonetheless, our study had limitations, including small sample size, short-term follow-up, and short treatment duration. DNS can be used to improve functional capacity in OW/OB individuals. In everyday clinical practice, it can be a useful tool to encourage more active participation from patients who have comorbidities, such as OB. More high-quality RCTs can be performed by subgrouping the individuals with OW/OB into a smaller range (as per BMI), thus helping researchers to see the actual effect of DNS.

## Conclusions

The aim of our research was to study the effect of DNS in OW/OB individuals on their functional capacity. BMI and SMW tests were used as the outcome measures. Both the control and experimental groups improved significantly from their baseline BMI and SMW test results, but when compared, the differences were not statistically significant. Both groups were found to be effective in increasing functional capacity in those who are OW/OB. The study indicates a strong need for further research into DNS’ long-term effectiveness in the OW/OB population.
